# Still I Croon

**DOI:** 10.1089/pmr.2020.0076

**Published:** 2021-06-24

**Authors:** Henry Bair

**Affiliations:** Stanford University School of Medicine, Stanford, California, USA.

**Keywords:** medical humanities, medical poetry, pain management

## Abstract

The is a poem inspired by my conversations with several patients burdened with chronic pain and who found themselves dependent on opioids for relief. For these patients, pain is an omnipresent and debilitating force permeating their existence, curbed only by the use of opioids; I was struck by the vivid language these patients would use when describing their relationship with pain and with these medications—some of which are directly used in this poem. They would simultaneously express a torn affection for the opioids, while recognizing the danger of continuing to use them. The poem is structured as an imaginary conversation between the patient/narrator and a personification of the medication/addiction. With this poem, I hope to share with readers a sense of the nuanced struggle and affliction I witnessed.

Pain is the background noise of my life;

it keeps a steady rhythm,

each heartbeat sending out pulsing

music I dance to

like a captive puppet on the strings

that force my feet to tap

to its involuntary measure.

*“Dance, my dear, dance for me,”*

the Voice whispers at first

then laughs out loud.

The song begins again.

I wince as I repeat

unrehearsed choreography

as the timpani rolls to its crescendo

and I collapse into a crying heap

upon the floor.

And still I croon—

Come to me, my White Angel.

Slip down my throat and enter my veins.

Hush the banging of the drums

and calm the wailing siren within.

Darkness falls and all the world's asleep,

but still I sense the beat.

I cover my ears, and my mouth opens wide

in a silent scream

that nobody hears. It reverberates

through empty chambers of my hollow life

and echoes back its mocking tune,

*“Dance, my lady, dance for me,”*

I lift my knees a little higher

and hope to appease my tormentor

who will not let me go.

But the laughs grow louder,

and trumpets join the beating drums

to blare their sinister psalm,

a cacophonous canticle

that will not let me rest.

And still I croon—

Come to me, my White Angel.

Tell me what you wish of me; tell me now,

and I shall do your bidding.

Take my wrists; I receive your shackles.

The Voice that once only whispered

a reminder of its presence

as it knocked upon the door

of each nerve ending,

has gained entrance at last

and made itself at home.

It licks its lips and leers, purring,

“*Dance, my lover, dance for me.*

*What will you give me?*

*What would you part with forever*

*to have me for a day?*

*Surrender the quietude of your home?*

*Spurn the embrace of your God?”*

I place my soul upon an altar

set before my idol in a bottle.

And still I croon—

Come to me, my White Angel,

my heart is empty, my spirit bare.

I know not how this story ends,

But you have triumphed for the day.

